# P-655. Validation Of DRIP Score And Derivation of Local Score Predicting Multi-Drug Resistant Pathogens in Patients with Community Acquired Pneumonia

**DOI:** 10.1093/ofid/ofae631.852

**Published:** 2025-01-29

**Authors:** Nayle Ibragimova, Emily A Siegrist, Joseph Sassine, Stephen Neely, Cindy B McCloskey, Bryan White

**Affiliations:** University of Oklahoma Health Sciences Center, Harker Heights, Texas; OU Health, Oklahoma City, Oklahoma; University of Oklahoma Health Sciences Center, Harker Heights, Texas; University of Oklahoma College of Pharmacy, Oklahoma City, Oklahoma; University of Oklahoma College of Medicine, Oklahoma City, OK; University of Oklahoma Medical Center, Oklahoma City, Oklahoma

## Abstract

**Background:**

A subset of patients with community acquired pneumonia (CAP) may develop pneumonia due to multi-drug resistant organisms (MDROs), and identifying risk factors for MDROs can be challenging. The 2019 CAP guideline recommends using “locally validated risk factors” to determine the need for empiric treatment of MDROs. The Drug Resistance in Pneumonia (DRIP) score is a commonly used tool to predict MDROs in CAP and was validated in various institutions. We performed this study to validate the performance of the DRIP score in our patient population and to derive a local risk prediction score.

**Figure:**
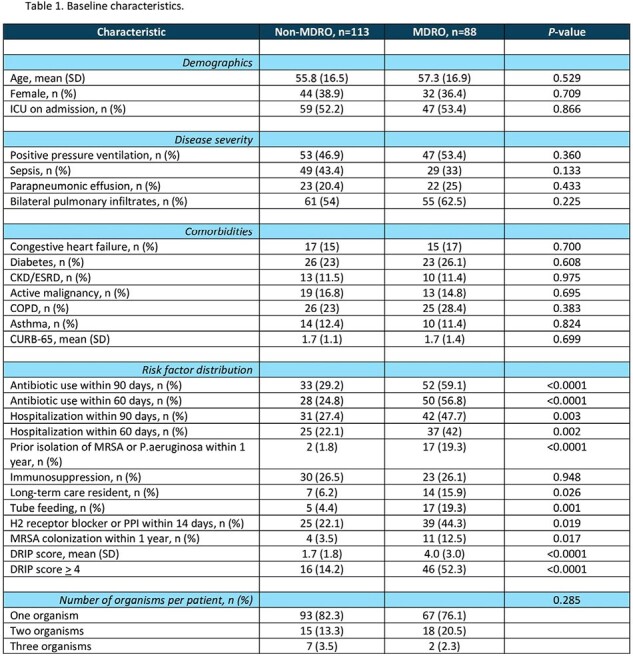

**Methods:**

This was a retrospective chart review of patients with positive cultures from respiratory samples, blood, or pleural fluid for organisms known to cause pneumonia with data obtained from March 1, 2021 to January 31, 2023. Data on disease severity, comorbidities, previous hospitalizations, prior antibiotic use, and history of infections with MDROs were used to locally validate the DRIP score, and to derive local risk prediction score using logistic regression.

**Figure:**
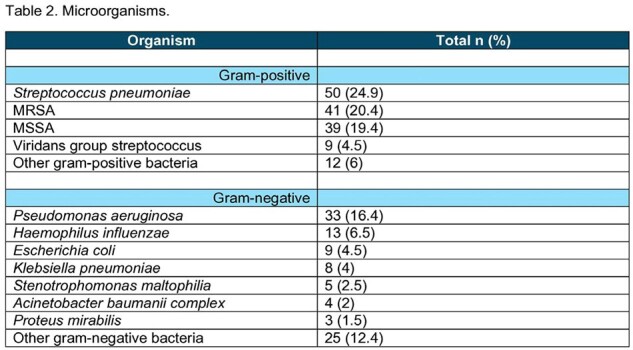

**Results:**

Utilizing data from 201 patients, the DRIP score demonstrated a sensitivity of 52%, specificity of 86%, positive predictive value (PPV) of 74%, and negative predictive value (NPV) of 70% for an MDRO as the microbiological etiology of CAP, using a threshold of > 4 points. Logistic regression analysis showed that history of infection with MDROs within 1 year, antibiotic use within 60 days, tube feeding, and chronic pulmonary disease were additional local risk factors in predicting CAP caused by an MDRO with a sensitivity of 49%, specificity of 86%, PPV of 73%, and NPV of 68%.

**Figure:**
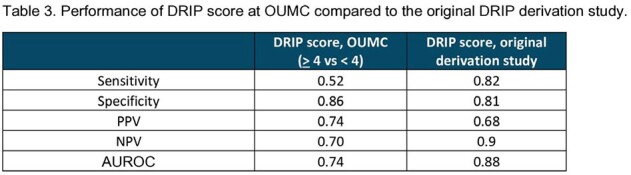

**Conclusion:**

At our institution the DRIP score performed with lower sensitivity and lower NPV than the original DRIP validation study. The locally derived score based on the above 4 risk factors also demonstrated decreased performance. These findings may represent limitations of retrospective design where previous antibiotic history, hospitalization, and laboratory data were not always available, resulting in false-positive and false-negative results. Additionally, a proportion of false-negative results occurred in patients who had a post-influenza or post-COVID-19 secondary bacterial pneumonia due to methicillin-resistant *Staphylococcus aureus*.

**Disclosures:**

**Joseph Sassine, MD**, Ansun BioPharma: Grant/Research Support|Cidara Therapeutics: Grant/Research Support|Community Infusion Solutions: Grant/Research Support|F2G: Grant/Research Support|Shionogi: Grant/Research Support **Bryan White, PharmD, BCPS, BCIDP**, Melinta: Advisor/Consultant|Shionogi: Grant/Research Support

